# Suspected heparin-induced thrombocytopenia in a COVID-19 patient on extracorporeal membrane oxygenation support: a case report

**DOI:** 10.1186/s12959-020-00252-9

**Published:** 2020-12-14

**Authors:** Xuan T. Phan, Tuan H. Nguyen, Tung T. Tran, Thu-Hien T. Huynh, Thuy-Ha T. Hoang, Vinh-Chau V. Nguyen, Thao N. T. Pham

**Affiliations:** 1 Intensive Care Unit, Cho Ray Hospital, Ho Chi Minh City, Vietnam; 2 Department of Hematology, Cho Ray Hospital, 201B Nguyen Chi Thanh Street, Ward 12, District 5, Ho Chi Minh City, Vietnam; 3 Hospital for Tropical Diseases in Ho Chi Minh City, Ho Chi Minh City, Vietnam

**Keywords:** HIT, ECMO, Extracorporeal, COVID-19, Thrombocytopenia

## Abstract

**Background:**

Extracorporeal membrane oxygenation (ECMO) support can be life-saving in critically ill COVID-19 patients. However, there are many complications associated with this procedure, including Heparin-induced thrombocytopenia (HIT.) Despite its rarity in ECMO cases, HIT can lead to devastating consequences and is difficult to manage.

**Case presentation:**

In this report, we present a case of a COVID-19 patient on ECMO support who was diagnosed with HIT and required intensive treatment. Initially, HIT was only suspected due to newly-developed thrombocytopenia and oxygenator dysfunction, with thrombi observed later. Regarding his treatment, since there was no recommended replacement to heparin available to us at the time of diagnosis, we decided to use rivaroxaban temporarily. No adverse events were recorded during that period. The patient was able to make a full recovery.

**Conclusion:**

HIT may jeopardize patient’s care during ECMO. As COVID-19 may bring about a surge in the number of patients requiring ECMO support, we need consented guidance to optimize treatment in this specific situation.

## Background

Coronavirus disease 2019 (COVID-19), the disease caused by Severe acute respiratory syndrome coronavirus 2 (SARS-CoV-2) infection, is an ongoing medical problem worldwide [1]. Patients may have different severity levels, ranging from mild dyspnea or coughing to multiorgan failure [1]. For those with life-threatening complications, intensive interventions may be requisite. Extracorporeal membrane oxygenation (ECMO) is a potentially life-saving procedure, in which the patient’s blood is circulated through an oxygenator to provide oxygen to vital organs [2]. In ECMO, the tubing system is usually coated with heparin to reduce to risk of thrombosis due to widespread coagulation activation throughout the set [2]. However, this practice gives rise to an increased risk of Heparin-induced thrombocytopenia (HIT), a condition in which platelets are incessantly activated by anti-PF4/Heparin antibodies, leading to catastrophic thrombotic events [2]. In this report, we describe a case of a COVID-19 patient on ECMO with suspected HIT who was particularly difficult to manage due to our lack of resources.

## Case report

A 43-year-old Caucasian male patient was admitted to the Hospital for Tropical Diseases in Ho Chi Minh City due to fatigue, mild fever, dry coughing and shortness of breath. He was diagnosed with SARS-CoV2 infection after his positive PCR result and was transferred to the isolation room. He was also given subcutaneous enoxaparin for venous thrombosis prophylaxis as part of COVID-19 treatment. After 4 days of enoxaparin administration, his condition quickly worsened and he required intubation and mechanical ventilation 2 weeks after admission, and veno-venous extracorporeal membrane oxygenation (ECMO) using ROTAFLOW pump (Maquet, Germany) and PLS-i oxygenator (Maquet, Germany), together with continuous renal replacement therapy (CRRT) 1 day later. Enoxaparin was stopped and unfractionated heparin (UFH) was given as a bolus dose of 8000 units, then 1200–1700 units/hour to maintain an activated partial thromboplastin time (aPTT) of 60–80 s. After the start of ECMO, he was routinely monitored by complete blood count (CBC) every 12–24 h, which revealed a drop is his platelet count to 44 × 10^9^/l one day after ECMO start, necessitating platelet transfusion. For the next few days, his platelet count continued to fluctuate. Furthermore, the patient required 2 oxygenator exchanges within 4 days after the start of ECMO due to increased transmembrane pressure. After the second exchange, several tests were performed to clarify his hypercoagulable status, including D-Dimer, antithrombin level, anti-β2-glycoprotein, anti-Cardiolipin, anti-PF4/Heparin antibodies, plasminogen, plasminogen activator inhibitor-1 (PAI-1), α2-antiplasmin quantification assays, as well as routine tests like aPTT, PT and fibrinogen level. All the assays were within normal ranges or negative, except for the anti-PF4/Heparin antibody test (HemosIL® HIT-Ab, Instrumentation Laboratory, Bedford, MA,) which showed a high antibody titer of 2.9 U/ml (normal range 0.0–1.0 U/ml), an elevated D-Dimer of 5.594 μg/ml and a prolonged aPTT of 53.1 s. One day later, there was a sudden drop in the flow volume from the outflow cannulae, noises of obstruction coming from the ECMO pump and thrombi were observed in the tubing by attending physicians. Coagulation assays were reassessed, showing a prolonged aPTT (87.7 s,) a normal fibrinogen level (3.65 g/L,) a high D-Dimer value of 6.050 μg/ml and a rising anti-PF4/Heparin antibody titer of 4.0 U/ml. As a result, HIT was suspected and UFH was stopped. However, we did not have access to other intravenous anticoagulants and the non-heparin coated disposables for ECMO at that moment. Therefore, we decided to start rivaroxaban given through the patient’s nasogastric tube at 15 mg twice daily and continued using the current ECMO set. An anti-Xa based assay (HemosIL Liquid anti Xa, Instrumentation Laboratory, Bedford, MA) was also performed 4 times per day to monitor the trough and peak rivaroxaban levels. At the same time, we added an HA280 immunoadsorption column (Jafron Biomedical Co., Ltd., China) to the CRRT system to filter out the antibodies. Anti PF4-Heparin antibody titer was undetectable 48 h after the start of rivaroxaban and we removed the hemoperfusion cartridge. The patient’s platelet count started to bounce back after 4 days of rivaroxaban use and fully recovered (> 150 × 10^9^/l) after 7 days. However, D-Dimer remained consistently high at 5.067–6.538 μg/ml throughout rivaroxaban administration. Nevertheless, no thrombotic event, including unexpected oxygenator exchange (besides fortnightly replacements recommended by the manufacturer,) was recorded during rivaroxaban use. After 10 days, we managed to get argatroban and switched to such anticoagulant. The patient required ECMO support with argatroban infusion for 42 more days. Throughout this period, his platelet count remained > 150 × 10^9^/l. Meanwhile, his D-Dimer was initially high at 5.0–7.0 μg/ml, with occasional peaks of > 10.0 μg/ml, usually accompanied by sudden increases in his interleukin-6 level, but then dropped to about 2.0–3.0 μg/ml when his overall condition improved. No thrombotic or hemorrhagic events were recorded during argatroban use. He was able to wean off ECMO and other mechanical supports and made a full recovery, with D-Dimer returning to normal 2 weeks after ECMO weaning. He was discharged on oral rivaroxaban for venous thrombosis prophylaxis, after more than 3 months of hospitalization.

**Fig. 1:**
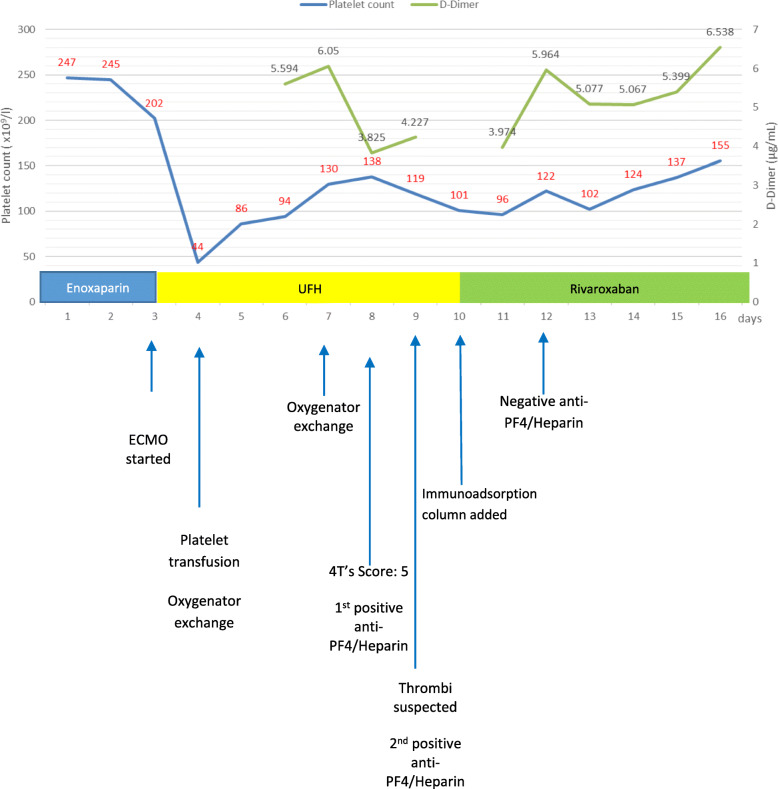
Summary of key laboratory results and treatment course

Key events in the patient’s treatment course and laboratory results are summarized in Fig. 1.

## Discussion and conclusion

Current reports have highlighted the hypercoagulable state of COVID-19 patients and its implication in the patients’ prognosis [1]. Consequently, the International Society on Thrombosis and Hemostasis (ISTH) have issued guidelines that recommend prophylactic Low-molecular-weight heparin for almost all COVID-19 patients [3]. However, this routine use of heparin may precipitate an increased risk of HIT, a potentially catastrophic thrombotic event, especially in critically ill patients. In patients on ECMO support, the incidence of HIT is reported to be 0.36% [2]. In critically ill COVID-19 patients, only a few cases have been described, with devastating consequences [4]. To evaluate the probability of HIT, physicians normally use a clinical assessment tool, such as the 4Ts Scoring system [2]. Our patient had a 4Ts score of 5 (Platelet count fall > 50% and nadir ≥20 × 10^9^/l: 2 points, consistent fall between 5 and 10 days, but unclear: 1 point, suspected thrombosis: 1 point, possible other causes of thrombocytopenia: 1 point,) suggesting an intermediate probability of HIT [5]. Nevertheless, the 4Ts score may be of little value in COVID-19, since there are many factors affecting its credibility. For example, in COVID-19, thrombocytopenia is a common finding and is linked to a plethora of etiologies [6]. To further complicate matters, thrombocytopenia can also be caused by other ECMO-related etiologies such as platelet activation and aggregation in the ECMO circuit, as well as multi-organ failure while on ECMO support [7]. The first drastic drop in our patient’s clinical course may be related to these issues, besides the presence of HIT antibodies, since his platelet count recovered after transfusion, without any clear sign of thrombus formation [7]. However, we should be aware that thrombosis may prove difficult to detect, partly due to the isolation requirements. Indeed, only 1 in the first 3 reported COVID-19 patients with HIT had typical clinical manifestations [4]. In our patient, a drop in platelet count which only improved after UFH cessation and oxygenator dysfunction were the initial signs of HIT. Actually, several reports have shown that thrombocytopenia and associated oxygenator dysfunction should prompt the suspicion of HIT in patients on ECMO support [8, 9]. To make a definitive diagnosis of HIT, an immunologic assay and a functional assay are required [10]. However, functional assays like the serotonin-releasing assay are time-consuming, difficult to perform and, as we encountered in our case, are not always available [10]. Immunologic tests are generally very sensitive and easy to perform, but not very specific [10]. Our patient had 2 separate positive results for anti-PF4/Heparin antibodies and matching clinical manifestations, thus his HIT diagnosis is highly likely. However, without results from a functional test, other hypercoagulable disorders could not be ruled out definitely. One possible differential diagnosis is sepsis-related disseminated intravascular coagulation (DIC.) Elevated D-Dimer, thrombocytopenia and thrombosis are common findings in DIC. However, D-Dimer are usually high in ECMO patients, [11] and can also be observed in cytokine release syndrome, a common complication of severe SARS-CoV-2 infection [12], while prolonged coagulation times are associated with anticoagulant use. Moreover, PAI-1, plasminogen, α2-antiplasmin, which are usually abnormal in sepsis-related DIC [13, 14], were within normal ranges. Therefore, DIC was unlikely in our patient.

While establishing the diagnosis of HIT in this patient was difficult, managing his condition was even more challenging. Firstly, it is recommended that when HIT is suspected, heparinoids should be stopped immediately and switching to another type of anticoagulants is warranted [2]. In ECMO, the preferred alternatives are intravenous anticoagulants such as bivalirudin, danaparoid, argatroban and fondaparinux [2, 15]. Unfortunately, none of the mentioned drugs was available to us at the time of HIT diagnosis. Consequently, we decided to start oral rivaroxaban, a factor Xa direct inhibitor. Although there have been reports regarding the efficacy and safety of rivaroxaban in patients with HIT [2], there is very little data about its use in the ECMO setting. From our experience, rivaroxaban was seemingly effective and safe, as we observed no thrombotic or bleeding events during our obligatory use of such drug. Besides the use of rivaroxaban, we also used an immunoadsorption column normally used in patients with autoimmune disorders to filter out antibodies, [16] in the hope that it can quickly remove the anti-PF4/Heparin antibodies from the patient’s circulation. Immunoadsorption has been used in various autoimmune disorders, usually in acute, life-threatening cases as it can rapidly remove harmful autoantibodies from the patient’s circulation [17, 18]. In HIT, this practice has not widely been described, except for a single case report, in which immunoadsorption helped remove the antibodies after just 3 days [19], while normally antibodies will only completely disappear about 3 months after heparin cessation [20]. In our case, after 2 days of immunoadsorption, the HIT antibody titer became undetectable. Therefore, immunoadsorption seems to be effective in HIT, especially when rapid removal of antibodies is required. Nevertheless, further studies are necessary to clarify the role of this promising therapy in HIT.

Another aspect of HIT management is whether to change the ECMO circuit or not. There are currently commercially available non-heparin coated tubing sets intent to use in HIT cases [9]. Nevertheless, many reports have showed that changing the ECMO circuit has no impact on the patients’ survival, since heparin in the tubing set cannot diffuse into the patients’ blood [15]. In our case, we did not, and admittedly could not, change the ECMO circuit, as there was no available alternative. However, we encountered no complication with continuous use of the heparin-coated tubing set.

The patient presented in this report is arguably the most complex COVID-19 case treated in Vietnam up to this date. His treatment was complicated by many affecting factors, most notably HIT. In general, management of HIT in patients on ECMO support is still difficult, as there is a lack of well-designed trials to clarify the importance of various practices in this setting. Our case highlights the need for consented guidelines in this specific situation, especially when COVID-19 is causing more and more patients to require life-saving ECMO support.

## Data Availability

Data sharing is not applicable to this article as no datasets were generated or analyzed during the current study.

## Abbreviations

aPTT: Activated partial thromboplastin time
CBC: Complete blood count
COVID-19: Coronavirus disease 2019
CRRT: Continuous renal replacement therapy
DIC: Disseminated intravascular coagulation
ECMO: Extracorporeal membrane oxygenation
HIT: Heparin-induced thrombocytopenia
ISTH: International Society on Thrombosis and Hemostasis
SARS-CoV-2: Severe acute respiratory syndrome coronavirus 2
UFH: Unfractionated heparin
